# Bones and Crohn's: Estradiol deficiency in men with Crohn's disease is not associated with reduced bone mineral density

**DOI:** 10.1186/1471-230X-8-48

**Published:** 2008-10-23

**Authors:** J Klaus, M Reinshagen, G Adler, BO Boehm, C von Tirpitz

**Affiliations:** 1 University of Ulm, Department of Internal Medicine I, Robert Koch Str. 8, 89081 Ulm, Germany; 2 Städtisches Klinikum Braunschweig, Department of Internal Medicine I, Salzdahlumer Straße 90, 38126 Braunschweig, Germany; 3 Medizinische Klinik, Kreisklinik Biberach, Ziegelhausstraße 50, 88400 Biberach, Germany

## Abstract

**Background:**

Reduced bone mineral density (BMD) and osteoporosis are frequent in Crohn's disease (CD), but the underlying mechanisms are still not fully understood. Deficiency of sex steroids, especially estradiol (E2), is an established risk factor in postmenopausal osteoporosis.

**Aim:**

To assess if hormonal deficiencies in male CD patients are frequent we investigated both, sex steroids, bone density and bone metabolism markers.

**Methods:**

111 male CD patients underwent osteodensitometry (DXA) of the spine (L1–L4). Disease related data were recorded. Disease activity was estimated using Crohn's disease activity index (CDAI). Testosterone (T), dihydrotestosterone (DHT), estradiol (E2), sex hormone binding globulin (SHBG), Osteocalcin and carboxyterminal cross-linked telopeptids (ICTP) were measured in 111 patients and 99 age-matched controls.

**Results:**

Patients had lower T, E2 and SHBG serum levels (p < 0.001) compared to age-matched controls. E2 deficiency was seen in 30 (27.0%) and T deficiency in 3 (2.7%) patients but only in 5 (5.1%) and 1 (1%) controls. Patients with E2 deficiency had significantly decreased T and DHT serum levels. Use of corticosteroids for 3 of 12 months was associated with lower E2 levels (p < 0.05). Patients with life-time steroids >10 g had lower BMD. 32 (28.8%) patients showed osteoporosis, 55 (49.5%) osteopenia and 24 (21.6%) had normal BMD. Patients with normal or decreased BMD showed no significant difference in their hormonal status. No correlation between markers of bone turnover and sex steroids could be found. ICTP was increased in CD patients (p < 0.001), and patients with osteoporosis had higher ICTP levels than those with normal BMD.

**Conclusion:**

We found an altered hormonal status – i.e. E2 and, to a lesser extent T deficiency – in male CD patients but failed to show an association to bone density or markers of bone turnover. The role of E2 in the negative skeletal balance in males with CD, analogous to E2 deficiency in postmenopausal females, deserves further attention.

## Background

Osteopenia and osteoporosis are frequently seen in Crohn's disease (CD) [1-7]. The prevalence of osteoporotic fractures is strikingly high, both in females and males [1,2,8-11]. However, pathogenesis and the risk factors indicative of an altered bone homeostasis are not fully understood. Most studies suggest a major impact of corticosteroid (CS) treatment on bone mineral density (BMD) but inflammation per se may exert an important risk since key inflammatory mediators such as the pro-inflammatory cytokines tumour necrosis factor-alpha (TNF-alpha), interleukin-1 beta (IL-1 beta) or IL-6 and other TNF-related cytokines such as receptor activator of nuclear factor kappa B (RANK) and its ligand, RANKL or osteoprotegerin are directly involved in the disease process [3,5-7,9,12-14].

In postmenopausal osteoporosis, the importance of sex steroid deficiency, especially estradiol (E2) deficiency, is well established [15-18]. Women with CD are at an increased risk of amenorrhoea and premature menopause [1]. Hormone replacement therapy (HRT) has been shown to be an effective treatment to prevent bone loss in postmenopausal women with CD [19].

Osteoporosis is often considered to be a disease of women, men lose half as much bone with aging and have one third as many fragility fractures as do women [15]. The regulation of bone metabolism involves a complex interplay between different factors, including sex steroids [15,17,18,20,21]. As of today, little is known about the prevalence of sex steroid deficiency in male CD patients and its contribution to bone loss and osteoporosis.

Previous studies investigating testosterone (T) and the gonadotropins follicle stimulating hormone (FSH) and luteinizing hormone (LH), reported lower than normal T levels in 4/48 (8%) male CD patients with the free androgen index (FAI) low in three patients and normal gonadotropins [22]. T was positively associated with osteocalcin [22]. Others report T deficiency in 20/45 (44.4%) male IBD patients but no effect on bone density and metabolism was detected [23]. Lower than normal dehydroepiandrosterone sulfate (DHEAS) levels have been found in IBD patients, in part dependent on previous glucocorticoid treatment [24,25]. High cortisol and low DHEAS levels were associated with higher humoral inflammatory activity, and vice versa [24]. Lower levels of DHEAS plasma levels in male IBD patients were associated with lower BMD and with higher deoxypyridinoline excretion. DHEAS correlated with BMD at the lumbar spine and femoral neck [23].

There are no data about estrogen (E), estradiol (E2) or the free estrogen index (FEI) and its association to bone loss and osteoporosis in men with CD.

Because E and T are the major sex steroids both in women and men, respectively, E was suggested to regulate bone turnover in women and T in men [20]. Loss of E after menopause is the single most important factor in development of postmenopausal bone loss and osteoporosis [15,18,21]. There is also growing evidence of a major role of estrogens in the regulation of bone metabolism in men [15,17,20,21,26]. Male patients with E resistance due to an oestrogen receptor missense mutation [27] or aromatase deficiency associated failure to convert androgens to estrogens [28,29] showed that E is important for optimal bone mass acquisition in young adulthood and for the retardation of bone loss in aging individuals [20]. Therefore, declining levels of E, especially estradiol (E2), may contribute to age-related bone loss in men suggesting that the male skeleton may require a threshold level of E for preventing increased bone resorption and bone loss [15,18,20,21,26,30,31].

Hormonal changes, especially altered E2 status, may therefore be a relevant factor for bone loss and osteoporosis in male patients with inflammatory bowel disease (IBD).

To assess if hormonal deficiencies in male CD patients are frequent and to address the role of these hormonal changes as relevant factor for bone loss and osteoporosis we investigated sex steroid serum levels, including E2, in 111 male CD patients and 99 age-matched male controls. In the 111 male CD patients, sex steroid serum levels were related to bone density and metabolism.

## Materials and methods

### Patients

111 consecutive male CD outpatients were included in the study. All patients had a prior diagnosis of Crohn's disease based on histological, endoscopic, radiological or clinical criteria. Disease related data on the previous and current state of health were recorded using a standardised questionnaire. Disease activity was estimated using the Crohn's disease activity index (CDAI) [32]. Nutritional status was assessed by body mass index (BMI). Cumulative life-time steroid dose was estimated and expressed in grams of prednisolone equivalent. Current CS use was defined as patients who have used CS for at least 3 of the past 12 months and patients who used CS at the date of the study.

Exclusion criteria included: (1) age less than 18 years, (2) chronic renal insufficiency (serum creatinine >1.5 mg/dl), (3) known primary hypo- or hyperparathyroidism, (4) untreated thyroid disease, (5) Paget's disease of bone, and any known medication or condition affecting bone density or sex hormone status other than glucocorticoids.

### Controls

The 99 age-matched male controls were recruited from healthy blood-donors who were not on medication or suffering from any known condition affecting bone density or sex hormone status.

### Ethical consideration

The study was approved by the Ethics Committee of the University of Ulm, Germany, (application number 1282000), and conducted in accordance with the 1975 Helsinki Declaration, as revised in 1983. All participants gave oral and written informed consent before inclusion.

### Laboratory testing

Blood samples were obtained in the morning, frozen and stored at -70°C until assayed. The sex hormone status was established in all subjects by measuring total testosterone [T; DSL-4000 ACTIVE™ Testosterone Radioimmunoassay, DSL GmbH Germany; intra- and interassay coefficients of variation (CVs) were 7.8–9.6% and 8.4–9.1%, respectively, sensitivity was 0.28 nmol/l, normal range: 9.7–30.5 ng/ml], dihydrotestosterone (DHT; DSL-9600 ACTIVE™ Radioimmunoassay, DSL GmbH Germany; CVs were 3.1–6.2% and 2.3–8.5%, respectively, sensitivity was 4.0 pg/ml, normal range: 94 – 476 pg/ml), estradiol (E2; DSL-39100 Radioimmunoassay, DSL GmbH Germany; CVs were 3.4–3.9% and 4.1–9.9%, respectively, sensitivity was 0.6 pg/ml, normal range: 21 – 41 pg/ml) and sex hormone binding globulin, the major high affinity sex steroids binding transport protein (SHBG; KP32CT Radioimmunoassay, DSL GmbH Germany; CVs were 4.2–5.9% and 4.6–5.9%, respectively, sensitivity was 2.5 nmol/ml, normal range 9 – 55 nmol/ml). The free androgen (FAI, testosterone/SHBG × 100) and estrogen (FEI, estradiol/SHBG × 100) index were calculated to estimate the bioavailable amount of T and E2. Serum osteocalcin (DSL-7600 ACTIVE™ Radioimmunoassay, DSL GmbH Germany; CVs were 1.4–4.6% and 3.2–5.8%, respectively, sensitivity was 0.3 ng/ml) and ICTP (ICTP Radioimmunoassay, Orion Diagnostica; CVs were 2.8–6.2% and 4.1–7.9%, respectively, sensitivity was 0.5 μg/ml, normal range: 1.3–5.2) were measured as markers of bone turnover, indicating bone formation and resorption. Normal reference ranges were provided by the manufacturers.

### Measurement of bone mineral density

Bone mineral density (BMD) of the lumbar spine (L1–L4) was assessed by DXA (Hologic QDR 1000, Hologic Inc., Waltham, MA). The average BMD values for L1–L4 were used for calculations. Normal values were supplied by Hologic Inc.. Assessment of BMD was performed according to the manufacturer's instructions. Bone mineral density (BMD) results were expressed as number of standard deviations (SD) from the peak bone mass of a young adult gender-matched reference population (T-score). According to WHO recommendation for postmenopausal women, osteopenia was defined as T-score < -1.0 SD, osteoporosis as T-score < -2.5 SD [33].

### Statistics

Results are given as median and range since data were not normally distributed. Qualitative variables are expressed as frequency (n) and percentage (%). For comparison of group means, the Mann Whitney non parametric rank test was used. Two-tailed tests for significance were used in all statistical analyses and p = 0.05 was considered statistically significant. Correlation coefficients were calculated by using Spearman's rank correlation test. Stepwise linear regression analysis was used to determine independent variables predictive for BMD. The Statistical Package SAS V6.11 was used for the analysis.

## Results

### • Baseline clinical characteristics

111 male CD patients were included in this study and compared to 99 age-matched healthy male controls. Table 1 provides baseline characteristics.

**Table 1:** Baseline characteristics

	Patients	Controls
Number	n = 111	n = 99
Age (yr)	35 (19 – 71)	38 (22 – 68)
Body mass index (kg/m^2^)	23.4 (16.8 – 34.7)	n.a.
T-score lumbar spine	-1.98 (-3.94 – 0.84)	n.a.
Patients with osteopenia (-2.5 T-score < -1)	55/49.6%	n.a.
Patients with osteoporosis (T-score < -2.5)	32/28.8%	n.a.
CDAI	104 (2 – 380)	n.a.
Duration of disease (yr)	8 (0.1 – 41.2)	n.a.
Maximal digestive extension:		
- small inflammatory bowel disease	42/37.8%	n.a.
- large inflammatory bowel disease	25/22.5%	n.a.
- small and large inflammatory bowel disease	44/39.6%	n.a.
Digestive resections	54/48.7%	n.a.
Lifetime corticosteroid dose:		
none	4/4.0%	n.a.
< 10 g	58/57.4%	n.a.
> 10 g	39/38.6%	n.a.
Corticosteroid users in 3 of the past 12 months	55/49.6%	n.a.
Current corticosteroid users	37/35.7%	n.a.
Current immunosuppressants (Azathioprine, 6-Mercaptopurine)	61/54.9%	n.a.
Current anti-TNF users	7/6.3%	n.a.

Data expressed as median with range shown in brackets. Qualitative data expressed as absolute values (n)/percentage.

### • Biochemical markers – Sex steroid serum levels

**Figure 1:**
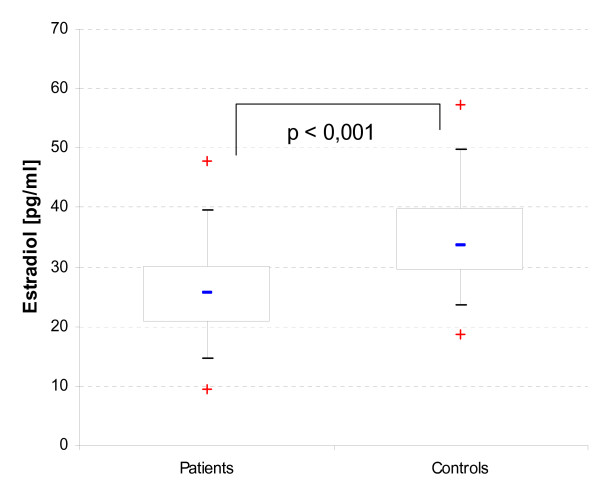
Estradiol (E2) serum levels, comparing male CD patients to healthy controls.

Compared to age matched controls, patients had significantly decreased serum levels of testosterone (T, 18.2 nmol/l vs. 21.4 nmol/l), estradiol (E2, 25.6 pg/ml vs. 33.7 pg/ml, see figure 1) and sex hormone binding globulin (SHBG, 21.0 nmol/l vs. 33.2 nmo/l; p < 0.001 each) (table 2). No significant difference could be observed comparing dihydrotestosterone (DHT) serum levels and the free estrogen index (FEI). The free androgen index (FAI) was higher in patients (87 vs. 65; p < 0.01) but 6 patients (two patients with osteoporosis) and only 2 controls had a FAI < 30. T deficiency (< 9.7 nmol/l) but normal FAI was seen in 3 (2.7%) patients and 1 control, with 2 of these 3 patients suffering from osteoporosis.

**Table 2:** Sex hormones and bone turnover markers in 111 male CD patients compared to 99 healthy controls

	**Patients**		**Controls**		
	(n = 111)		(n = 99)		
	Median	Range	Median	Range	p

Testosterone [nmol/l]	18.2	8.8 – 48	21.4	8.7 – 39.6	*
SHBG [nmol/l]	21.0	6.9 – 69.3	33.2	6.9 – 70.5	*
FAI	87	18 – 351	65	27 – 208	**
DHT [pg/ml]	269.5	66 – 750	279.5	80 – 798	n.s.
Estradiol [pg/ml]	25.6	9.4 – 47.8	33.7	18.7 – 57.4	*
FEI	45	10 – 140	40	10 – 160	n.s.
Osteocalcin [ng/ml]	3.0	0.3 – 12.8	2.5	0.3 – 14.9	n.s.
ICTP [μg/l]	3.8	1.7 – 20.8	3.3	2.0 – 7.1	*

* p < 0,001; ** p < 0,01

E2 serum levels below the normal range (< 21 pg/ml) were seen in 30 (27.0%) patients including 14 patients with osteoporosis but in only 5 (5.1%) controls. Patients with E2 deficiency had significantly lower serum levels of T, DHT, FAI and FEI and a higher disease activity (CDAI) compared to patients with normal E2 serum levels (table 3).

**Table 3:** 30 male CD patients with vs. 81 male CD patients without estradiol (E2) deficiency

	**Estradiol < 21 pg/ml**		**Estradiol > 24 pg/ml**		
	(n = 30)		(n = 81)		
	Median	Range	Median	Range	p

Age (yr)	33.0	19 – 68	38.5	19 – 71	n.s.
CDAI	176	6 – 380	88	2 – 354	**
Testosterone [nmol/l]	18.2	8.8 – 29.7	19.8	10.8 – 48.0	***
SHBG [nmol/l]	21	7.6 – 65.6	21	6.9 – 69.3	n.s.
FAI	63	21 – 184	95	18 – 351	*
DHT [pg/ml]	179	66 – 479	297	90 – 750	***
FEI	32	9 – 83	53	17 – 143	****
Osteocalcin [ng/ml]	3.4	0.5 – 8.9	2.9	0.3 – 12.8	n.s.
ICTP [μg/l]	4.0	1.9 – 20.8	3.8	1.7 – 11.7	n.s.
T-score spine	-2.06	-3.94 – 0.4	-1.96	-3.94 – 0.84	n.s.

* p < 0,05; ** p < 0,01; *** p < 0,001; **** p < 0,0001

Patients with a regimen including CS for at least 3 of the past 12 months before the study had lower E2 (24.7 pg/ml vs. 26.7 pg/ml; p < 0.05) and T (16.1 nmol/l vs. 20.6 nmol/l; p < 0.01) serum levels compared to patients who had not used CS. The difference in E2 and T serum levels of current CS users and CS non-users did not reach statistical significance.

With age, T and FAI decreased in both, patients (T: r = -0.24, p < 0.01; FAI: r = -0.27, p < 0.01) and controls (FAI: r = -0.34, p < 0.001), while SHBG serum levels increased. E2 and FEI decreased in controls but not in patients (r = -0.33, p < 0.001 and r = -0.28, p < 0.001, respectively), but patients had lower E2 levels in all groups of age (table 4).

**Table 4:** Comparing age stratified cohorts of male CD patients vs. healthy male controls

	**< 30 years**			**30 – 45 years**			**> 45 years**		
	**Patients** **(n = 32)**	**Controls** **(n = 31)**	**p**	**Patients** **(n = 48)**	**Controls** **(n = 36)**	**p**	**Patients** **(n = 31)**	**Controls** **(n = 32)**	**p**

Testosterone (nmol/l)	20.1 (8.8 – 40.6)	23.0 (11.2 – 33.9)		18.7 (9.6 – 48.0)	21.7 (10.4 – 39.6)	0.025	15.5 (11 – 26.2)	19.4 (8.7 – 32.8)	< 0.01
SHBG (nmol/l)	19,3 (6.9 – 47.3)	32 (8.2 – 46.9)	<0.0025	21.4 (11.5 – 69.3)	28.3 (6.9 – 70.5)		24.1 (12.2 – 52.7)	37.8 (12.7 – 70.4)	< 0.01
FAI	100 (34 – 100)	78 (39 – 163)	<0.025	86 (18 – 232)	65.5 (28 – 208)		67 (26 – 146)	52.5 (27 – 168)	
DHT (pg/ml)	278 (66 – 562)	310 (95 – 616)	<0.05	263 (67 – 750)	266 (103 – 712)		262 (110 – 750)	218 (80 – 798)	
Estradiol (pg/ml)	25.8 (12.2 – 39.6)	37.5 (29.8 – 57.4)	<0.001	24.8 (9.4 – 46.6)	32.1 (20.1 – 45.6)	<0.001	26.7 (18.4 – 47.8)	30.9 (18.7 – 52.6)	<0.0025
FEI	49 (15 – 140)	53 (25 – 136)		40 (9 – 93)	45 (15 – 158)		39 (14 – 143)	36 (11 – 96)	
Osteocalcin (ng/ml)	3.4 (1.0 – 12.8)	4.5 (1.0 – 14.9)		2.8 (0.5 – 10.1)	2.4 (0.6 – 6.0)		2.6 (0.3 – 9.5)	2.1 (0.3 – 5.4)	
ICTP (μg/l)	4.4 (2.1 – 11.7)	3.9 (2.0 – 7.1)	<0.05	3.6 (1.7 – 20.8)	3.1 (2.2 – 4.7)	<0.0025	3.6 (2.3 – 6.0)	3.2 (2.0 – 4.8)	<0.025
T-score (lumbar spine)	-1.72 (-3.8 – +0.05)			-2.02 (-3.9 – +0.84)			-2.11 (-3.94 – +0.28)		

Patients with active (CDAI > 150) compared to non-active disease (CDAI < 150) had significantly lower FEI (40 vs. 50; p < 0.025). Higher BMI was associated with higher E2 and FEI levels (table 5).

**Table 5:** Sex hormones and bone turnover markers of 111 male CD patients divided in subgroups according to Body mass index (BMI)

	**A**		**B**		**C**		
	**BMI < 20 **(n = 26)		**BMI 20–25 **(n = 55)		**BMI > 25 **(n = 30)		p
	Median	Range	Median	Range	Median	Range	

Testosterone (nmol/l)	19.3	8.8 – 34.4	20.4	9.5 – 48	16,95	11.1–37.1	^a)^
SHBG (nmol/l)	25.6	7.6 – 69.3	22.2	8.5 – 57	17,15	6.9 – 65.1	^b)c)^
FAI	61.5	19 – 351	90	26 – 338	93	18 – 236	n.s.
DHT (pg/ml)	256	66 – 643	270	84 – 643	275	147 – 750	n.s.
Estradiol (pg/ml)	21.6	14.1 – 41.5	25.5	9.4 – 46.6	27.1	15.3 – 47.8	^d)e)^
FEI	35	11 – 129	41	9 – 140	57	16 – 143	^f)g)h)^
Osteocalcin (ng/ml)	3.5	0.7 – 12.1	3.1	0.5 – 12.8	2.7	0.3 – 7.6	n.s.
ICTP (μg/l)	4.9	2.8 – 11.7	3,7	2.1 – 8.3	3.5	1.7 – 5.6	^i)j)^
lumbar T-score	-2.36	-3.94 – -0.25	-2.06	-3.94–0,4	-1.56	-3.27–0.84	^k)l)^
Age (yr)	30.5	19 – 47	34	21 – 71	45	26 – 66	^m)n)^

^a) ^p < 0,05 group B vs. C; ^b) ^p < 0,02 group A vs. C; ^c) ^p < 0,05 group B vs. C; ^d) ^p < 0,05 group A vs. B; ^e) ^p = 0,005 group A vs. C;^f) ^p = 0,05 group A vs. B; ^g) ^p < 0,001 group A vs. C; ^h) ^p < 0,01 group B vs. C;^i) ^p < 0,01 group A vs. B; ^j) ^p < 0,001 group A vs. C; ^k) ^p < 0,025 group A vs. C;^l) ^p < 0,05 group B vs. C.^m) ^p < 0,001 group A vs. C.^n) ^p < 0,01 group B vs. C.

### • Bone mineral density

The mean lumbar BMD T-score of the 111 CD patients was -1.98 ± 1.06. 32 patients (28.8%) showed osteoporosis, 55 (49.5%) osteopenia and 24 (21.6%) patients had normal lumbar BMD (table 6).

**Table 6:** Sex hormones and bone turnover markers of 111 male CD patients according to bone mineral density (BMD)

	**normal BMD**		**Osteopenia**		**Osteoporosis**		
	(n = 24)		(n = 55)		(n = 32)		
	Median	Range	Median	Range	Median	Range	p

Age (yr)	41	19 – 67	32	19 – 63	40	19–71	n.s.
BMI (kg/m^2^)	25.1	19.7–34.7	23.0	16.8–29.4	22,4	16.9–30.8	*
Testosterone [nmol/l]	18.8	11.1–34.4	17.3	9.5 – 40.6	18.1	8.8 – 48	n.s.
SHBG [nmol/l]	20.6	6.9 – 57	22.2	8.5 – 69.3	18.2	7.6 – 65.6	n.s.
FAI	86.5	26 – 351	79	18 – 338	96	23 – 232	n.s.
DHT [pg/ml]	275	119 – 590	269	67 – 750	260	66 – 750	n.s.
Estradiol [pg/ml]	26.5	14.2–38.4	26.1	9.4 – 47.8	24.8	14.2–41.5	n.s.
FEI	50	10 – 130	40	10 – 140	40	10 – 111	n.s.
Osteocalcin [ng/ml]	2.6	0.7 – 10.1	2.9	0.5 – 12.8	3.3	0.3 – 12.1	n.s.
ICTP [μg/l]	3.5	1.9 – 20.8	3.7	1.7 – 11.6	4.2	2.7 – 11.7	*

* p < 0,05 comparing patients with normal BMD to patients with osteoporosis

Patients with a life-time steroid dose >10 g had a lower lumbar BMD (T-score -2.39 ± 1.03 vs. -1.71 ± 0.98, p < 0.005). BMD and BMI were interrelated, patients with the lowest BMI < 20 having the lowest BMD (table 5). BMD decreased with age in CD patients (table 4).

In the linear regression analysis, the only significant independent predictors for BMD of the lumbar spine were age (p = 0.04), BMI (p = 0.002) and life-time steroid dose > 10 g (p = 0.007). These variables together accounted for 19% of BMD T-score.

### • Biochemical markers – Sex steroids and bone mineral density

No significant differences in the sex steroid serum levels could be detected comparing the sub-groups of patients with normal lumbar BMD, osteopenia or osteoporosis, respectively (table 6) and no difference in lumbar BMD of patients with vs. without E2 deficiency was seen (table 3). There was no correlation between sex steroids and BMD, neither in all the 111 CD patients nor in any sub-group.

### • Biochemical markers of bone turnover and sex steroids/bone mineral density

ICTP serum levels were increased in patients compared to controls (3.8 μg/l vs. 3.3 μg/l; p < 0.001). Significantly increased ICTP could be detected in the sub-group with osteoporosis compared to patients with normal BMD (4.2 μg/l vs. 3.5 μg/l; p < 0.05). Patients with BMI < 20 kg/m^2 ^had significant higher ICTP levels than patients with BMI > 20 kg/m^2 ^(table 5). In patients with higher inflammatory activity, indicated by CRP levels > 5 mg/l, bone turnover rates were increased with higher ICTP (4.3 μg/l vs. 3.6 μg/l; p < 0.025) and osteocalcin serum levels (3.5 ng/ml vs. 2.8 ng/ml; p < 0.05). No association between sex steroid serum levels and the bone metabolism markers osteocalcin and ICTP was found.

Regarding the 111 male CD patients according to age at the time of diagnosis (younger vs. older than 21 years), no significant differences in sex steroid serum levels nor bone mineral density were found.

## Discussion

Crohn's disease (CD) is a multisystem disorder characterised by chronic intestinal inflammation. This disorder often leads to reduced bone mineral density (BMD) and osteoporosis.

To understand the role of endocrine factors CD associated osteoporosis we investigated sex steroid serum levels, including E2, in 111 male CD patients and 99 age-matched male controls.

In our study, T and SHBG serum levels were decreased in patients compared to controls (table 2). T deficiency (< 9.7 nmol/l) but normal FAI was seen in only 3/111 (2.7%) patients and 1/99 (1%) controls. No correlation between T or SHBG and BMD or markers of bone turnover could be found. These results suggest that T deficiency is an uncommon finding in male CD patients, confirming data reported before [22,23,34].

Alterations of androgen (A) serum levels are believed to play a role in male osteoporosis, but the relationship between T serum levels and bone loss in men is less well documented compared to E loss in postmenopausal osteoporosis [15,18]. Ageing otherwise healthy men lack a distinct hormonal equivalent of the female menopause with T serum levels not changing significantly over life [15,18,21,35] and in the majority of cases, no overt hypogonadism in men with osteoporosis is seen [18,26,30,35,36]. Only the FAI, what is the serum bio-available not SHBG bound T, decreases in elderly men due to an age related increase in SHBG serum levels [17,35,37,38].

Two studies reported T deficiency in 4/48 (8%) and 20/45 (44.4%) male IBD patients with 1 and 15 of them on CS therapy, respectively [22,23]. T had no effect on bone density and metabolism [23]. Lower than normal FAI was reported in 3/48 (6.2%) CD patients but FAI was only calculated in men with low or borderline testosterone [22].

There are no data about E2 or FEI and its association to bone loss and osteoporosis in men with CD.

In our study, E2 serum levels but not the FEI were significantly decreased in patients. Lower than normal E2 serum levels (< 21 pg/ml) were more present in CD patients (30/111; 27.0%) versus controls (5/99; 5.1%). Patients with E2 deficiency had significantly decreased T, DHT, FAI and FEI serum levels and a higher disease activity (CDAI) (table 3, figure 1). CS usage for at least 3 of the past 12 months had decreased E2 serum levels. However, no correlation between E2 serum levels or FEI and bone density or markers of bone turnover could be observed.

Some studies reported a closer interrelation between BMD and estrogen compared to BMD and androgen status in males [15,20,30]. The FEI had a higher predictive value for BMD at all skeletal sites [34] in both, young (<50 years) and older (>50 years) men [17,30,31,35]. E2 serum levels were decreased but within the normal range in male idiopathic osteoporotic patients [30], suggesting that the skeleton may require a certain threshold level of E to prevent increased bone resorption and bone loss [18,20,21,26,31]. There is little or no change in E2 serum levels over life in men but similarly to FAI, the FEI, what is the serum bio-available not SHBG bound E2, decreases in elderly men due to the age related increase in SHBG serum levels [17,21,31,38].

In CD both, reduced bone formation and increased bone resorption is reported to play a central role in bone loss and osteoporosis [2,4,5]. Reduced DHEAS levels in male IBD patients were associated with higher deoxypyridinoline excretion [23] and T was positive associated with osteocalcin [22]. Therefore, estrogen deficiency is expected to increase bone turnover rates [18,35].

Our results confirm a higher bone resorption rate in CD patients. CD patients with osteoporosis or BMI < 20 kg/m^2 ^revealed the highest ICTP serum levels. If the association of decreased ICTP serum levels in CD patients with BMI > 20 kg/m^2 ^and higher E2/FEI serum levels due to an altered E2/FEI ratio remains unclear because we were not able to show an association with markers of bone turnover.

The prevalence of osteoporosis (28.8%), osteopenia (49.5%) and normal BMD (21.6%) in men with CD was comparable to prevalence rates reported before [1,2,4-7]. A life-time steroid dose > 10 g was associated with lower lumbar BMD, confirming former reports [3,5-7,9,12,13]. Likewise to glucocorticoids CS therapy induces a dose-dependent increase in risk of fracture and BMD loss [12]. Patients with BMI < 20 kg/m^2 ^had a lower lumbar BMD compared to patients with a higher BMI, confirming previous reports that a lean body habitus seems to be a risk factor for osteoporosis in CD patients [5,7,30,35,37].

The multiple regression model generated for BMD explained 19% of the variance by the parameters age, BMI and life-time steroid dose, parameters which have been reported before [1-3,5,7]. The models R^2 ^value suggests that other factors may contribute significantly to BMD. Genetic factors are reported to be most important, with up to 60 – 80% of BMD variance being genetically determined, so only a smaller part may be determined by other factors including hormonal status [18].

Several cross-sectional studies on idiopathic osteoporosis in men demonstrated a closer relation between E and BMD compared to T and BMD [17,20,30,31,36]. However, in our study, sex steroids were not significant different in patients with osteoporosis vs. patients with osteopenia or normal BMD, respectively (table 6). No association of BMD to/with T, E2 or FEI serum levels was found, neither in all patients nor in any sub-group. This probably indicates that bone metabolism markers and sex steroids compared to BMD taken at one time reflect different processes in time with indices like BMD not changing rapidly compared to humoral markers. BMD may be the cumulative result of different processes taking place earlier and over a longer period of time. Only data of a follow-up study relating rates of change in BMD to serum sex steroids levels would prove the effect of E and T deficiency on bone loss and osteoporosis as confounding factor associating BMD and sex steroids also in men with CD.

## Conclusion

Our results show that male CD patients have decreased E2 and, to a lesser extent T serum levels with nearly one-third of male CD patients having E2 serum levels below the normal range. We could not consider low E2 levels as a surrogate marker for glucocorticoid treatment or disease activity in general, even with regard to the results that male CD patients with higher disease activity, according to CDAI, or CS users had decreased E2/FEI and T serum levels. Overall our data emphasize the fact that an optimal control of inflammation in CD patients is crucial.

We failed to show a direct interrelation between the altered hormonal status and bone density and metabolism, confirming results in primary male osteoporosis, where E2 deficiency in 14/42 (33%) men did not correlate with BMD [26]. The role of E2 in the negative skeletal balance in males with CD, analogous to E2 deficiency in postmenopausal females, deserves further attention.

## Abbreviations

A: androgen; BMD: bone mineral density; CD: Crohn's disease; CDAI: Crohn's disease activity index; CS: corticosteroid; DHEAS: dehydroepiandrosterone sulphate; DHT: dihydrotestosterone; DXA: dual energy X-ray absorptiometry; E: oestrogen; E2: estradiol; FAI: free androgen index; FEI: free estrogen index; HRT: hormone replacement therapy; IBD: inflammatory bowel disease; ICTP: carboxyterminal cross-linked telopeptids; SHBG: sex hormone binding globulin; T: testosterone; WHO: World Health Organisation.

## Competing interests

None of the contributing authors has any financial or non-financial competing interests influencing interpretation of data or presentation of information.

## Authors' contributions

JK, CvonT contributed to conception and design, acquisition, analysis and interpretation of data, drafted and revised the manuscript; MR, GA, and BOB contributed to conception and design and revised the manuscript critically for important intellectual content; all authors have given final approval of the version to be published.

## Pre-publication history

The pre-publication history for this paper can be accessed here:



## References

[B1] Compston JE, Judd D, Crawley EO, Evans WD, Evans C, Church HA, Reid EM, Rhodes J (1987). Osteoporosis in patients with inflammatory bowel disease. Gut.

[B2] Abitbol V, Roux C, Chaussade S, Guillemant S, Kolta S, Dougados M, Coutourier D, Amor B (1995). Metabolic bone assessment in patients with inflammatory bowel disease. Gastroenterology.

[B3] Bernstein CN, Seeger LL, Sayre JW, Anton PA, Artinian L, Shanahan F (1995). Decreased bone density in inflammatory bowel disease is related to corticosteroid use and not disease diagnosis. J Bone Miner Res.

[B4] Bjarnason I, Macpherson A, Mackintosh C, Buxton-Thomas M, Forgacs I, Moniz C (1997). Reduced bone density in patients with inflammatory bowel disease. Gut.

[B5] von Tirpitz C, Pischulti G, Klaus J, Rieber A, Brueckel J, Boehm BO, Adler G, Reinshagen M (1999). Pathological bone density in chronic inflammatory bowel disease – prevalence and risk factors. Z Gastroenterol.

[B6] Tobias JH, Sasi MR, Greenwood R, Probert CS (2004). Rapid hip bone loss in active Crohn's disease patients receiving short term corticosteroid therapy. Aliment Pharmacol Ther.

[B7] Siffiledeen JS, Fedorak RN, Siminoski K, Jen H, Vaudan E, Abraham N, Steinhart H, Greenberg G (2004). Bones and Crohns disease: risk factors associated with low bone mineral density in patients with Crohns disease. Inflamm Bowel Dis.

[B8] Klaus J, Armbrecht G, Steinkamp M, Brueckel J, Rieber A, Adler G, Reinshagen M, Felsenberg D, von Tirpitz C (2002). High prevalence of osteoporotic vertebral fractures in patients with Crohn's disease. Gut.

[B9] Van Staa TP, Cooper C, Brusse LS, Leufkens H, Javaid MK, Arden NK (2003). Inflammatory bowel disease and the risk of fracture. Gastroenterology.

[B10] Pooran N, Singh P, Bank S (2003). Crohn's disease and risk of fractures: does thyreoid disease play a role?. World J Gastroenterol.

[B11] Loftus EV, Achenbach SJ, Sandborn WJ, Tremaine WJ, Oberg AL, Melton LJ (2005). Risk of fracture in ulcerative colitis: a population-based study from Olmsted County, Minnesota. Clin Gastroenterol Hepatol.

[B12] Van Staa TP, Leufkens HGM, Cooper C (2002). The Epidemiology of Corticosteroid-Induced Osteoporosis: a Meta-analysis. Osteoprosis Int.

[B13] von Tirpitz C, Epp S, Klaus J, Mason R, Hawa G, Brinskelle-Schmal N, Hofbauer LC, Adler G, Kratzer W, Reinshagen M (2003). Effect of systemic glucocorticoid therapy on bone metabolism and the osteoprotegerin system in patients with active Crohns disease. Eur J Gastroenterol Hepatol.

[B14] Tilg H, Moschen AR, Kaser A, Pines A, Dotan I (2008). Gut, inflammation and osteoporosis: basic and clinical concepts. Gut.

[B15] Riggs BL, Khosla S, Melton LJ (1998). A unitary model for involutional osteoprosis: estrogen deficiency causes both type I and type II osteoporosis in postmenopausal women and contributes to bone loss in ageing men. J Bone Miner Res.

[B16] Ettinger B, Pressman A, Sklarin P, Bauer DC, Cauley JA, Cummings SR (1998). Associations between low levles of serum estradiol, bone density, and fractures among elderly women: the study of osteoporotic fractures. J Clin Endocrinol Metab.

[B17] Khosla S, Melton LJ, Atkinson EJ, O'Fallon WM, Klee GG, Riggs BL (1998). Relationship of sex steroid levels and bone turnover markers with bone mineral density in men and women: a key role for bioavailable estrogen. J Clin Endocrinol Metab.

[B18] Compston JE (2001). Sex Steroids and Bone. Physiological reviews.

[B19] Clements D, Compston JE, Evans WD, Rhodes J (1993). Hormone replacement therapy prevents bone loss in patients with inflammatory bowel disease. Gut.

[B20] Khosla S, Melton LJ, Riggs BL (2001). Estrogens and Bone Health in Men. Calcif Tissue Int.

[B21] Khosla S, Melton LJ, Riggs BL (2002). Clinical review 144: Estrogen and the male skeleton. J Clin Endocrinol Metab.

[B22] Robinson RJ, Iqbal SJ, Al-Azzawi F, Abrams K, Meaberry JF (1998). Sex hormon status and bone metabolism in men with Crohn's disease. Aliment Pharmacol Ther.

[B23] Szathmári M, Vásárhelyi B, Treszl A, Tulassay T, Tulassay Z (2002). Association of dehydroepiandrosterone sulfate and testosterone deficiency with bone turnover in men with inflammatory bowel disease. Int J Colorectal Dis.

[B24] Straub RH, Vogl D, Gross V, Lang B, Scholmerich J, Andus T (1998). Association of humoral markers of inflammation and dehydroepiandrosterone sulfate or cortisol serum levels in patients with chronic inflammatory bowel disease. Am J Gastroenterol.

[B25] de la Torre B, Hedman M, Befrits R (1998). Blood and tissue dehydroepiandosterone sulphate levels and their relationship to chronic inflammatory bowel disease. Clin Exp Rheumatol.

[B26] Carlsen CG, Soerensen TH, Eriksen EF (2000). Prevalence of low serum estradiol levels in male Osteoporosis. Osteoporosis Int.

[B27] Smith EP, Boyd J, Frank GR, Takahashi H, Cohen RM, Specker B, Williams TC, Lubahn DB, Korach KS (1994). Estrogen resistance caused by a mutation in the estrogen-receptor gene in a man. N Engl J Med.

[B28] Conte FA, Greenbach MM, Ito Y, Fisher CR, Simpson ER (1994). A syndrome of female pseudohermaphroditism, hypergonadotropic hypogonadism, and multicystic ovaries associated with missense mutations in the gene encoding aromatase (P450arom). J Clin Endocrinol Metab.

[B29] Morishima A, Grumbach MM, Simpson ER, Fisher C, Qin K (1995). Aromatase deficiency in male and female siblings caused by a novel mutation and the physiological role of estrogen. J Clin Endocrinol Metab.

[B30] Gillberg P, Johansson AG, Ljunghall S (1999). Decreased estradiol levels and free androgen index and elevated sex-hormone binding globulin levels in male idiopathic osteoporosis. Calcif Tissue Int.

[B31] Pottelbergh I, Goemaere S, Kaufman JM (2003). Bioavailable Estradiol and an Aromatase Gene Polymorphism are Determinants of Bone Mineral Density Changes in Men over 70 Years of Age. J Clin Endocrinol Metab.

[B32] Best WR, Becktel JM, Singleton JW, Kern F (1976). Development of a Crohn's disease activity index. National co-operative Crohn's disease study. Gastroenterology.

[B33] WHO Study Group (1994). Assessment of fracture risk and its application to screening for postmenopausal osteoporosis. World Health Organ Tech Rep Ser.

[B34] Katznelson L, Fairfield WP, Zeizafoun N, Sands BE, Peppercorn MA, Rosenthal DI, Klibanski A (2003). Effects of Growth Hormone Secretion on Body Composition in Patients with Crohn's Disease. J Clin Endocrinol Metab.

[B35] Evans SF, Davie MWJ (2002). Low body size and elevated sex-hormone binding globulin distinguish men with idiopathic vertebral fracture. Calcf Tissue Int.

[B36] Slemenda CW, Longcope C, Zhou L, Hui SL, Peacock M, Johnston CC (1997). Sex steroids and bone mass in older men: positive association with serum estrogens and negative association with androgens. J Clin Invest.

[B37] Rapado A, Hawkins F, Sobrinho L, Diaz-Curiel M, Galvao-Telles A, Arver S, Melo Gomes J, Mazer N, Garcia e Costa J, Horcajada C, Lopez Gavilanes E, Mascarenhas M, Papapietro K, Lopez Alvarez MB, Pereira MC, Martinez G, Valverde I, Garcia JJ, Carballal JJ, Garcia I (1999). Bone Mineral Density and Androgen Levels in Elderly Males. Calcif Tissue Int.

[B38] Clarke BL, Ebeling PR, Jones JD, Wahner HW, O'Fallon WM, Riggs BL, Fitzpatrick LA (2002). Predictors of Bone Mineral Density in Aging Healthy Men Varies by Skeletal Site. Calcif Tissue Int.

